# Magnetic resonance spectroscopic correlates of progression free and overall survival in “glioblastoma, IDH-wildtype, WHO grade-4”

**DOI:** 10.3389/fnins.2023.1149292

**Published:** 2023-06-29

**Authors:** Banu Sacli-Bilmez, Ayça Erşen Danyeli, M. Cengiz Yakicier, Fuat Kaan Aras, M. Necmettin Pamir, Koray Özduman, Alp Dinçer, Esin Ozturk-Isik

**Affiliations:** ^1^Institute of Biomedical Engineering, Bogazici University, Istanbul, Türkiye; ^2^Department of Pathology, School of Medicine, Acıbadem Mehmet Ali Aydinlar University, Istanbul, Türkiye; ^3^Center for Neuroradiological Applications and Research, Acıbadem Mehmet Ali Aydinlar University, Istanbul, Türkiye; ^4^YoctoSensum Biotechnology, Istanbul, Türkiye; ^5^Department of Neuropathology, University of Heidelberg, Heidelberg, Germany; ^6^Department of Neurosurgery, School of Medicine, Acıbadem Mehmet Ali Aydinlar University, Istanbul, Türkiye; ^7^Department of Radiology, School of Medicine, Acıbadem Mehmet Ali Aydinlar University, Istanbul, Türkiye

**Keywords:** IDH-wildtype glioblastoma, TERTp mutation, glioma, survival, magnetic resonance spectroscopy, machine learning

## Abstract

**Background:**

The 2021 World Health Organization (WHO) Central Nervous System (CNS) Tumor Classification has suggested that isocitrate dehydrogenase wildtype (IDH-wt) WHO grade-2/3 astrocytomas with molecular features of glioblastoma should be designated as “Glioblastoma, IDH-wildtype, WHO grade-4.” This study analyzed the metabolic correlates of progression free and overall survival in “Glioblastoma, IDH-wildtype, WHO grade-4” patients using short echo time single voxel ^1^H-MRS.

**Methods:**

Fifty-seven adult patients with hemispheric glioma fulfilling the 2021 WHO CNS Tumor Classification criteria for “Glioblastoma, IDH-wildtype, WHO grade-4” at presurgery time point were included. All patients were *IDH1/2*-wt and *TERT*p-mut. ^1^H-MRS was performed on a 3 T MR scanner and post-processed using LCModel. A Mann–Whitney U test was used to assess the metabolic differences between gliomas with or without contrast enhancement and necrosis. Cox regression analysis was used to assess the effects of age, extent of resection, presence of contrast enhancement and necrosis, and metabolic intensities on progression-free survival (PFS) and overall survival (OS). Machine learning algorithms were employed to discern possible metabolic patterns attributable to higher PFS or OS.

**Results:**

Contrast enhancement (*p* = 0.015), necrosis (*p* = 0.012); and higher levels of Glu/tCr (*p* = 0.007), GSH/tCr (*p* = 0.019), tCho/tCr (*p* = 0.032), and Glx/tCr (*p* = 0.010) were significantly associated with shorter PFS. Additionally, necrosis (*p* = 0.049), higher Glu/tCr (*p* = 0.039), and Glx/tCr (*p* = 0.047) were significantly associated with worse OS. Machine learning models differentiated the patients having longer than 12 months OS with 81.71% accuracy and the patients having longer than 6 months PFS with 77.41% accuracy.

**Conclusion:**

Glx and GSH have been identified as important metabolic correlates of patient survival among “IDH-wt, TERT-mut diffuse gliomas” using single-voxel ^1^H-MRS on a clinical 3 T MRI scanner.

## Introduction

A fraction of lower-grade hemispheric diffuse gliomas in adults exhibit a more malignant clinical behavior (Brat et al., 2015; Eckel-Passow et al., 2015; Akyerli et al., 2018). Molecular genetic studies have clearly shown that most of these cases lack isocitrate dehydrogenase (*IDH1/2*) mutations, and carry molecular features of glioblastoma (Brat et al., 2015). The presence of the *TERT*-promoter (*TERT*p) mutation, *EGFR* gene amplification, or the combination of chromosome-7 gain and chromosome-10 loss (+7/−10 pattern) have gained wide acceptance as key molecular markers for worse outcome within IDH-wildtype (IDH-wt) glioblastoma (Reuss et al., 2015). Recently, the 2021 WHO Central Nervous System (CNS) Tumor Classification has suggested that gliomas with the aforementioned characteristics, should be designated simply as “Glioblastoma, IDH-wt, WHO grade-4” (Louis et al., 2021). The criteria suggested in the 2021 WHO CNS Tumor Classification are the presence of microvascular proliferation, necrosis or at least one of the three molecular alterations, which are TERTp mutation, EGFR gene amplification and + 7/−10 pattern (Louis et al., 2021). This redefinition and regrouping effectively has outlined high-risk gliomas across all histopathological grades. Since this is a relatively new definition, imaging markers that may provide more information on the biology of “IDH-wt, TERTp-mut diffuse gliomas” have not been fully understood yet.

Proton magnetic resonance spectroscopy (^1^H-MRS) is a valuable tool for studying the metabolic profiles of diffuse gliomas. Previously, *TERT*p-mut adult diffuse gliomas were identified among *IDH*-wt gliomas with 93% accuracy using ^1^H-MRS (Ozturk-Isik et al., 2019). The goal of this study is to identify the metabolic correlates of progression free survival (PFS) and overall survival (OS) in the “IDH-wt, TERTp-mut diffuse gliomas” that satisfy the criteria for IDH-wt glioblastoma using clinical short echo time (TE) single voxel ^1^H-MRS with machine learning.

## Materials and methods

### Patients

This was a retrospective analysis of data from a single institution. All patients underwent surgery at the Department of Neurosurgery, Acıbadem Mehmet Ali Aydınlar University School of Medicine between September 2011 and February 2019. Only adult patients with supratentorial, hemispheric diffuse *IDH1/2*-wt gliomas carrying one of the two *TERT*p hotspot mutations (C228T or C250T), identified on molecular analysis of the solid tumor, and who underwent preoperative diagnostic ^1^H-MRS were included. Thalamic, brainstem, cerebellar, spinal, recurrent, and pediatric cases (aged<18 years) were excluded. The final cohort included 57 (*IDH*-wt, *TERT*p-mut) adult patients with hemispheric diffuse gliomas (35 men/22 women, mean age: 58 ± 10.47 years, median: 59 years, range: 39–75 years). All the patients were followed up for up to 65 months, and their overall and progression free survival times were recorded. The study protocol was approved by the Institutional Review Board of Acıbadem Mehmet Ali Aydınlar University School of Medicine, and written informed consent was obtained from all patients, who approved the use of their data for research.

### Pathology and molecular genetics

The histopathological grade was determined by a single neuropathologist (AED, 12 years of experience) (Hoshide and Jandial, 2016). Histological grading was performed based on the presence of mitosis, vascular endothelial proliferation, necrosis, and IDH and TERTp mutations. One case that had necrosis on magnetic resonance imaging (MRI) but no histopathological examination was considered to have a WHO 2016 grade-4 tumor. The cohort included 45 (79%) patients with histological WHO 2016 grade-4 tumors, eight (14%) patients with WHO 2016 grade-3 tumors, and four (7%) patients with WHO 2016 grade-2 tumors. Molecular testing for *IDH1*(R132) and *IDH2*(R140 and R172) and *TERTp* (C228T and C250T) hotspot mutations was performed in all cases at the time of diagnosis using mini-sequencing and/or Sanger sequencing, as previously described (Akyerli et al., 2018).

### MRS acquisition and analysis

The patients underwent MRS 1–7 days before surgery using a clinical 3 T MR scanner (Siemens, Erlangen-Germany) and a standard brain tumor MR imaging protocol that included pre- and post-contrast (gadolinium DTPA) T1-weighted TSE (repetition time [TR] = 500 ms, TE = 10 ms), post-contrast T1-weighted volumetric TurboFLASH (TR = 2000 ms, TE = 4 ms), post-contrast T2-weighted TSE (TR = 5,000 ms, TE = 105 ms), and T2*-weighted gradient-echo echo-planar imaging dynamic susceptibility contrast MRI (TR = 1,500 ms, TE = 30 ms). ^1^H-MRS data were acquired from the solid tumor region excluding necrosis, edema, and hemorrhage using a point resolved spectroscopy (PRESS) sequence (TR/TE = 2000/30 ms, 1,024 points, 1,200 Hz, voxel size = 1–8 cm^3^, number of signal averages = 192, acquisition time = 6.5 min). Hemorrhage and necrosis were determined from pre- and post-contrast T1-weighted MRI scans. Since ^1^H-MRS data acquisition close to the air/tissue interface or scalp results in shimming difficulties, magnetic susceptibility artifacts, and lipid contamination, the voxel was placed as far away from these regions as possible. Contrast enhancement and necrosis were determined from the post-contrast T1-weighted MRI by an experienced radiologist with more than 30 years of experience (AD) to evaluate their effects on the survival of IDH-wt, TERTp-mut gliomas. The percentage of the extent of resection was calculated by comparing the pre-and post-operative segmented tumor volumes on axial T2-weighted MRI.

### Data processing

A basis set of 19 metabolites including creatine (Cr), phosphocreatine (PCr), gamma-aminobutyric acid, glutamine (Gln), glutamate (Glu), glycine (Glyc), glycerophosphocholine (GPC), phosphocholine (PCh), glutathione (GSH), 2-hydroxyglutarate (2HG), myo-inositol (mIns), lactate (Lac), N-acetyl aspartate (NAA), N-acetylaspartylglutamic acid (NAAG), and five composite peak concentrations (total choline [tCho = GPC + PCh], total creatine [tCr = Cr + PCr], total NAA [tNAA = NAA + NAAG], glutamine–glutamate complex [Glx], and myoinositol and glycine [mIns + Glyc]) were used to quantify the concentrations of the metabolites in the LCModel spectral fitting program (Provencher, 2001). The basis spectra were simulated using the chemical shifts and coupling constants provided by Govindaraju et al. (2001). In the LCModel control file, which is provided in the Appendix, the ppm window range and some data acquisition parameters (hzppm, echot, nunfill and deltat) were defined. In addition to metabolite concentrations in arbitrary units, metabolite to tCr ratios were also assessed. The basis set also included alanine, aspartate, glucose, scyllo-inositol, and taurine; however, these metabolites could not be quantified in most patients and were not included in further analysis. The signal to noise ratio (SNR) is defined as the ratio of the maximum in the spectrum-minus-baseline over the analysis window to twice the root mean square of residuals in LCModel, while FWHM (full width at half-maximum) is a rough estimate of the linewidths of the *in vivo* spectrum. Any spectrum with an SNR < 4 and a FWHM of >0.12 ppm was excluded from further analysis. A Cramer–Rao lower bound threshold of >30% was used, and the metabolites estimated with higher CRLB values were assigned “not a number.” The correlation matrix of quantification was created in LCModel, and negative correlations (≤-0.5) between the metabolites, which indicates coupled quantification due to their similar peak patterns, were investigated. Additionally, Pearson correlation coefficients were computed between the metabolites identified as correlated by LCModel. The coupled metabolites were excluded from the Cox multivariate regression and classification analyses.

### Statistical analysis and classification

A Mann–Whitney U test was used to assess the metabolic differences between “IDH-wt, TERTp-mut diffuse gliomas” with and without contrast enhancement and between those with and without radiological necrosis. Bonferroni multiple comparison correction was applied, and a *p*-value of ≤0.003 was considered as statistically significant for metabolic differences. The effects of contrast enhancement, necrosis, extent of resection, and metabolite concentrations on OS and PFS of “IDH-wt, TERTp-mut diffuse gliomas” were evaluated using Kaplan–Meier survival analysis, followed by a log-rank test. Associations between survival outcomes and different clinical predictors (age and sex), imaging features (the presence of contrast enhancement and necrosis), extent of resection, and metabolite concentrations were assessed using univariate and multivariate Cox regression models. Only the significant univariate parameters were included in the multivariate Cox regression analysis. Classification and regression tree (CART) analysis was used to calculate the predictive power of these variables on OS and PFS. Then, supervised machine learning algorithms were employed to identify IDH-wt, TERTp-mut gliomas with PFS of longer than 6 months and OS of longer than 12 months. A baseline model was developed based on clinical features, including age, contrast enhancement, necrosis and extent of resection for classification of IDH-wt, TERTp-mut gliomas with high PFS or OS. We also created classification models using the features selected between the metabolite concentrations as well as age, extent of resection, contrast enhancement and necrosis. Feature selection with the least absolute shrinkage and selection operator (Lasso) (Tibshirani, 1996), random forest recursive feature elimination (RFRFE) (Granitto et al., 2006), or sequential floating forward selection (SFFS) (Pudil et al., 1994) was applied before machine learning-based classification to eliminate redundant features and prevent overfitting. Fifteen models derived from well-known machine learning algorithms, such as k nearest neighbor (KNN), support vector machines (SVM), and ensemble trees, were used to classify IDH-wt, TERTp-mut gliomas with high PFS or OS based on their metabolic profiles. Five-fold cross-validation was used, and the models were executed 100 times to report the mean performance measures. The synthetic minority oversampling technique (SMOTE) (Chawla et al., 2002) was also employed to overcome the class imbalance problem in our classification. SMOTE was implemented in Python, CART analysis was performed using SPSS statistics 25 (IBM Corp., Armonk, NY), and the remaining computations were performed using MATLAB 2022a (MathWorks, Natick, MA).

## Results

### Cohort characteristics

Table 1 shows the characteristics of the study participants, including the pathological group and WHO 2016 grade, age, sex, tumor location, contrast enhancement and necrosis. Most tumors occurred in the frontal lobe region (19), followed by the temporal lobe (12), and the insular lobe (10). Gliomatosis cerebri growth pattern were observed only in two patients. Contrast enhancement was observed in 2/12 (17%) patients with WHO 2016 grade-2/3 tumors and 44/45 (98%) patients with WHO 2016 grade-4 tumors. Necrosis occurred in 43/45 (96%) patients with WHO 2016 grade-4 tumors. The median value for extent of resection was 61% [range = 10–100%]. Additionally, the extent of resection was higher than 95% in only seven patients.

**Table 1:** The clinical features of IDH-wt, TERTp-mut glioma patients included in this study.

Characteristics	Total (*n* = 57)
WHO 2016 histopathological grade (*n*, %)	
Grade-2	4 (7%)
Grade-3	8 (14%)
Grade-4	45 (79%)
Age at diagnosis (years)	
(Mean ± SD)	58 ± 10.47
(Median, range)	59 (39–75)
Sex (M/F)	35/22
Tumor location no. (*n*, %)	
Frontal lobe	19 (33%)
Parietal lobe	6 (11%)
Temporal lobe	12 (21%)
Insular region	10 (18%)
Occipital lobe	8 (14%)
Gliomatosis cerebri pattern (*n*, %)	2 (3%)
Contrast enhancing tumors (*n*, %)	46 (81%)
Necrosis (*n*, %)	43 (75%)
Extent of resection (%)	
(Median, range)	61 (10–100)

### LCModel analysis performance

**Figure 1:**
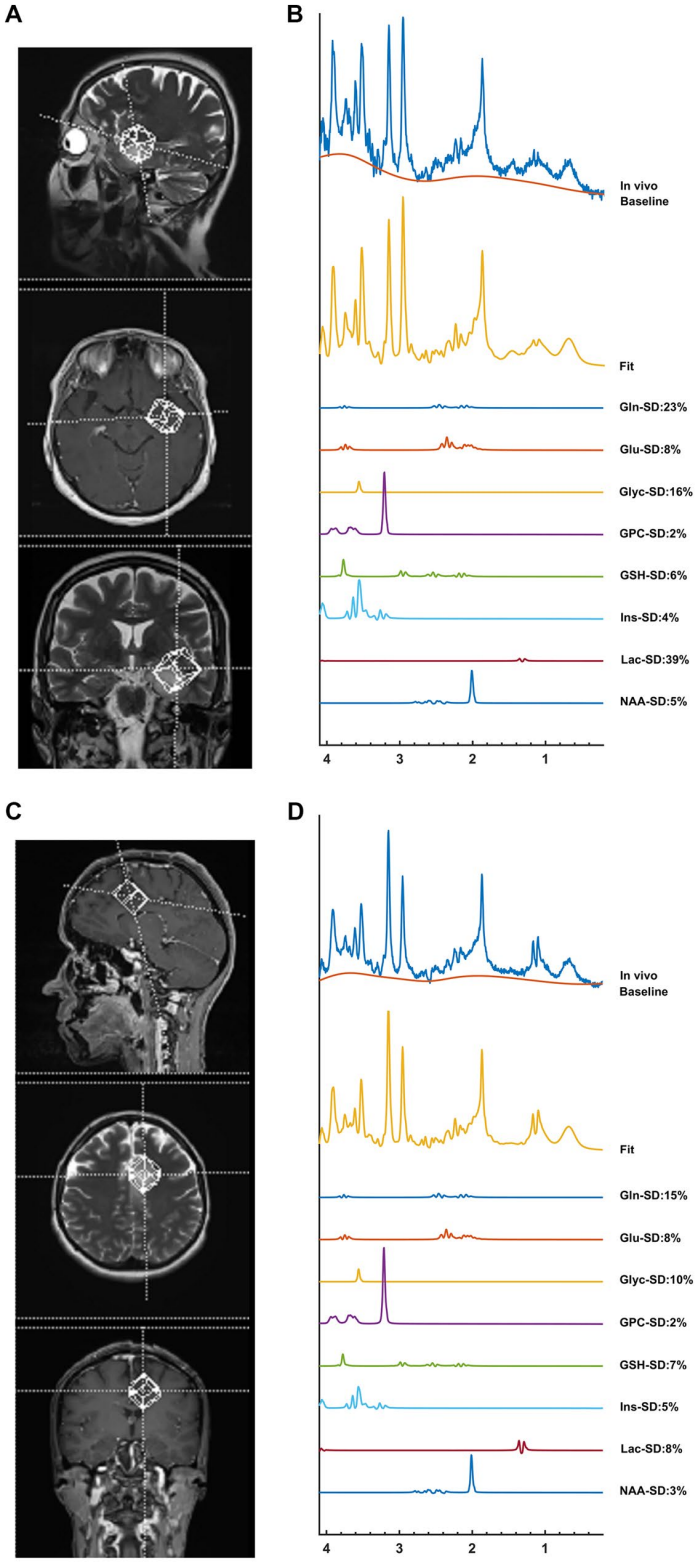
An example IDH-wt TERTp-mut glioma without contrast enhancement (top) and an example of IDH-wt TERTp-mut glioma with contrast enhancement (bottom). The voxel selection on T2-weighted MRI **(A,C)**, and MR spectroscopic data with some of the LCModel results **(B,D)**. While high mIns and GSH were visible in non-contrast enhancing tumor, high GPC and Lac were prominent in contrast enhancing tumor.

The average maximum SNR was 18.36 ± 14.43 and the average FWHM was 0.06 ± 0.02 ppm for the spectra of this patient population. Supplementary Figure S1 shows the mean correlation matrix output of the LCModel averaged over all patients. The correlation coefficient was <−0.5 for Cr and PCr in 39 patients (*r* = −0.64, *p* < 0.001) and for Glyc and mIns in 35 patients (*r* = −0.52, *p* < 0.001). The Pearson correlation coefficients were computed between the overlapping metabolites, which were −0.82 (*p* < 0.001) for Cr and PCr and −0.70 (*p* < 0.001) for Glyc and mIns. 2HG/tCr and NAAG/tCr could not be determined in more than 30% of the patients in each group, and these metabolites were excluded from further analysis. Additionally, Cr-PCr, Glu-Gln, GPC-PCh, and NAA-NAAG were coupled metabolites, so their composite values were included in the Cox multivariate regression analysis and the classification. Figure 1 shows short-TE PRESS MRS data along with the LCModel quantification results of two example gliomas.

### Effect of contrast enhancement

Contrast-enhanced tumors had significantly higher Gln/tCr (*p* = 0.002), GPC/tCr (*p* = 0.002), tCho/tCr (*p* < 0.001), and Glx/tCr (*p* < 0.001) levels than non-contrast-enhanced tumors (Supplementary Figure S2A). In addition, there was a trend for higher Glu/tCr (*p* = 0.009) and GSH/tCr (*p* = 0.007) (Supplementary Figure S2A).

### Effect of necrosis

Tumors with radiologically determined necrosis had higher Gln/tCr (*p* < 0.001), Glu/tCr (*p* < 0.001), GPC/tCr (*p* = 0.003), GSH/tCr (*p* < 0.001), Lac/tCr (*p* < 0.001), tCho (*p* < 0.001), and Glx (*p* < 0.001) levels than the other tumors (Supplementary Figure S2B).

Supplementary Figure S3 shows an additional spider plot visualization of the results presented in Supplementary Figure S2.

### Survival analysis

**Figure 2:**
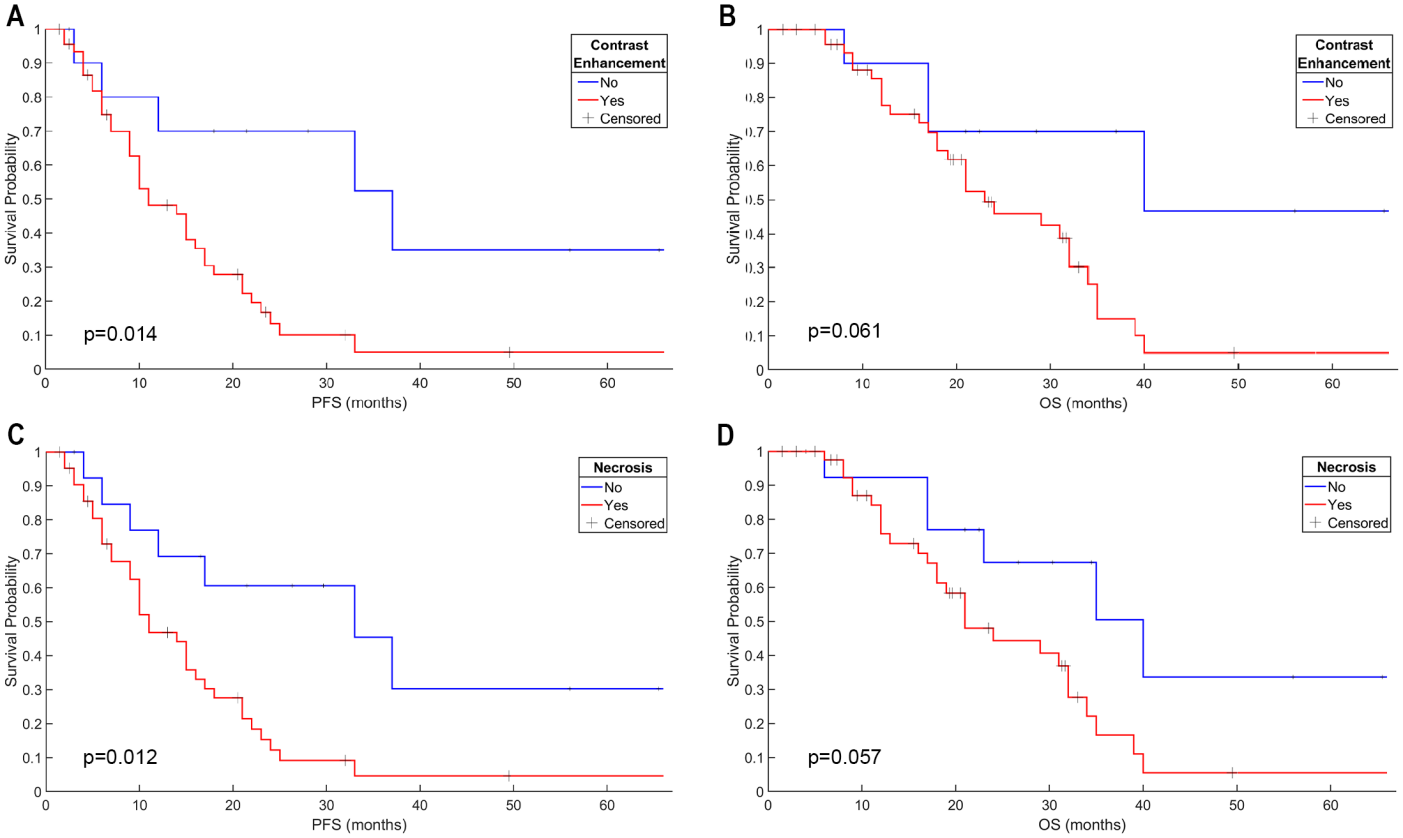
Contrast enhancement had a significant effect on PFS; however, there was no significant effect on OS of IDH-wt, TERTp-mut gliomas **(A,B)**. Necrosis had a significant effect on PFS; however, there was no significant effect on OS of IDH-wt, TERTp-mut gliomas **(C,D)**.

**Figure 3:**
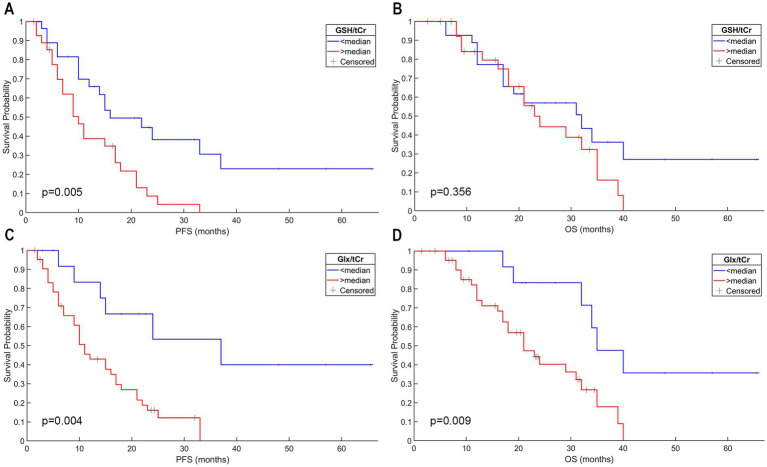
IDH-wt, TERTp-mut gliomas with higher GSH/tCr levels had a shorter PFS; however, the GSH/tCr level was not associated with worse OS in this patient group **(A,B)**. Higher Glx/tCr ratio was associated with worse PFS and OS in IDH-wt, TERTp-mut gliomas **(C,D)**.

The anatomical localization was not statistically significantly associated with either PFS or OS by using a log-rank test (*p* = 0.212 for PFS and *p* = 0.192 for OS) or univariate Cox regression analysis (*p* = 0.949 for PFS; *p* = 0.465 for OS). On the other hand, both contrast enhancement and necrosis were associated with significantly shorter PFS (*p* = 0.014 and *p* = 0.012, respectively, log-rank test; Figures 2A,C). However, contrast enhancement and necrosis were not found to be associated with a shorter OS (*p* = 0.061 and *p* = 0.057, respectively, log-rank test; Figures 2B,D). The Kaplan–Meier analysis also showed that a higher Glx/tCr measurement was associated with a significantly shorter PFS (*p* = 0.032) and a significantly shorter OS (*p* = 0.047; Figures 3A,B). A higher GSH/tCr ratio was also associated with a significantly shorter PFS (*p* = 0.004; Figures 3C,D). The median PFS and OS of WHO 2016 grade-2/3 tumors were 18 and 22.5 months, while those of WHO 2016 grade-4 tumors were 10 and 19 months, respectively. WHO 2016 grade-2/3 tumors had significantly longer PFS (*p* = 0.027; log-rank test) than WHO 2016 grade-4 tumors; however, there was no statistically significant difference in OS between the two groups (*p* = 0.057; log-rank test).

Univariate Cox model analysis using nine metabolic parameters as well as age, sex, contrast enhancement, and radiological necrosis indicated that six parameters were associated with a shorter PFS, which were the contrast enhancement (*p* = 0.015); presence of necrosis (*p* = 0.012); and higher levels of Glu/tCr (*p* = 0.007), GSH/tCr (*p* = 0.019), tCho/tCr (*p* = 0.032), and Glx/tCr (*p* = 0.010) (Table 2). Univariate Cox model analysis also revealed that the presence of necrosis (*p* = 0.049); and higher levels of Glu/tCr (*p* = 0.039), and Glx/tCr (*p* = 0.047) were associated with a significantly shorter OS (Table 2). On the other hand, multivariate Cox model analyses identified no factors that were significantly associated with OS or PFS (Table 3).

**Table 2:** Univariate Cox analysis for the association between survival outcomes and different predictors including clinical variables, imaging features, and metabolite concentrations.

Covariates	Progression-free survival			Overall survival		
	Coefficient (b)	Hazard ratio exp(b)	Value of *p*	Coefficient (b)	Hazard ratio exp(b)	Value of *p*
Age	0.007	1.007	0.652	0.017	1.018	0.310
Gender	−0.317	0.728	0.335	−0.171	0.843	0.637
Contrast Enhancement	1.211	3.358	0.015*	1.054	2.869	0.053
Necrosis	1.071	2.918	0.012*	0.906	2.474	0.049*
Extent of resection (%)	−0.006	0.995	0.236	−0.009	0.991	0.068
Gln/tCr	0.298	1.348	0.064	0.192	1.212	0.291
Glu/tCr	0.391	1.479	0.007*	0.334	1.397	0.039*
GPC/tCr	0.41	1.507	0.374	0.819	2.267	0.126
PCh/tCr	0.828	2.289	0.151	0.203	1.225	0.762
GSH/tCr	0.896	2.449	0.019*	0.743	2.102	0.096
2HG/tCr	0.092	1.096	0.449	−0.034	0.966	0.815
mIns/tCr	−0.445	0.641	0.205	−0.161	0.851	0.649
Lac/tCr	0.028	1.029	0.512	0.056	1.058	0.210
NAA/tCr	−0.469	0.626	0.300	−0.619	0.538	0.224
NAAG/tCr	0.062	1.064	0.930	0.293	1.34	0.661
tCho/tCr	1.002	2.724	0.032*	0.87	2.387	0.092
tNAA/tCr	−0.156	0.856	0.712	−0.095	0.909	0.819
mIns+Glyc/tCr	0.075	1.078	0.709	0.306	1.358	0.123
Glx/tCr	0.232	1.261	0.010*	0.195	1.215	0.047*

PCr + Cr = tCr, PCho + GPC = tCho, Glu + Gln = Glx, NAA + NAAG = tNAA.

*p ≤ 0.05.

**Table 3:** Multivariate Cox analysis for the association between survival outcomes and different predictors including clinical variables, imaging features, histological grade, and metabolite concentrations.

Covariates	Progression-free survival			Overall survival		
	Coefficient (b)	Hazard ratio exp(b)	Value of *p*	Coefficient (b)	Hazard ratio exp(b)	Value of *p*
Contrast enhancement	0.780	2.182	0.376	–	–	–
Necrosis	0.346	1.413	0.634	0.700	2.022	0.159
GSH/tCr	0.604	1.829	0.431	–	–	–
tCho/tCr	−0.46	0.633	0.601	–	–	–
Glx/tCr	0.075	1.077	0.636	0.120	1.129	0.268

### Machine learning results

**Figure 4:**
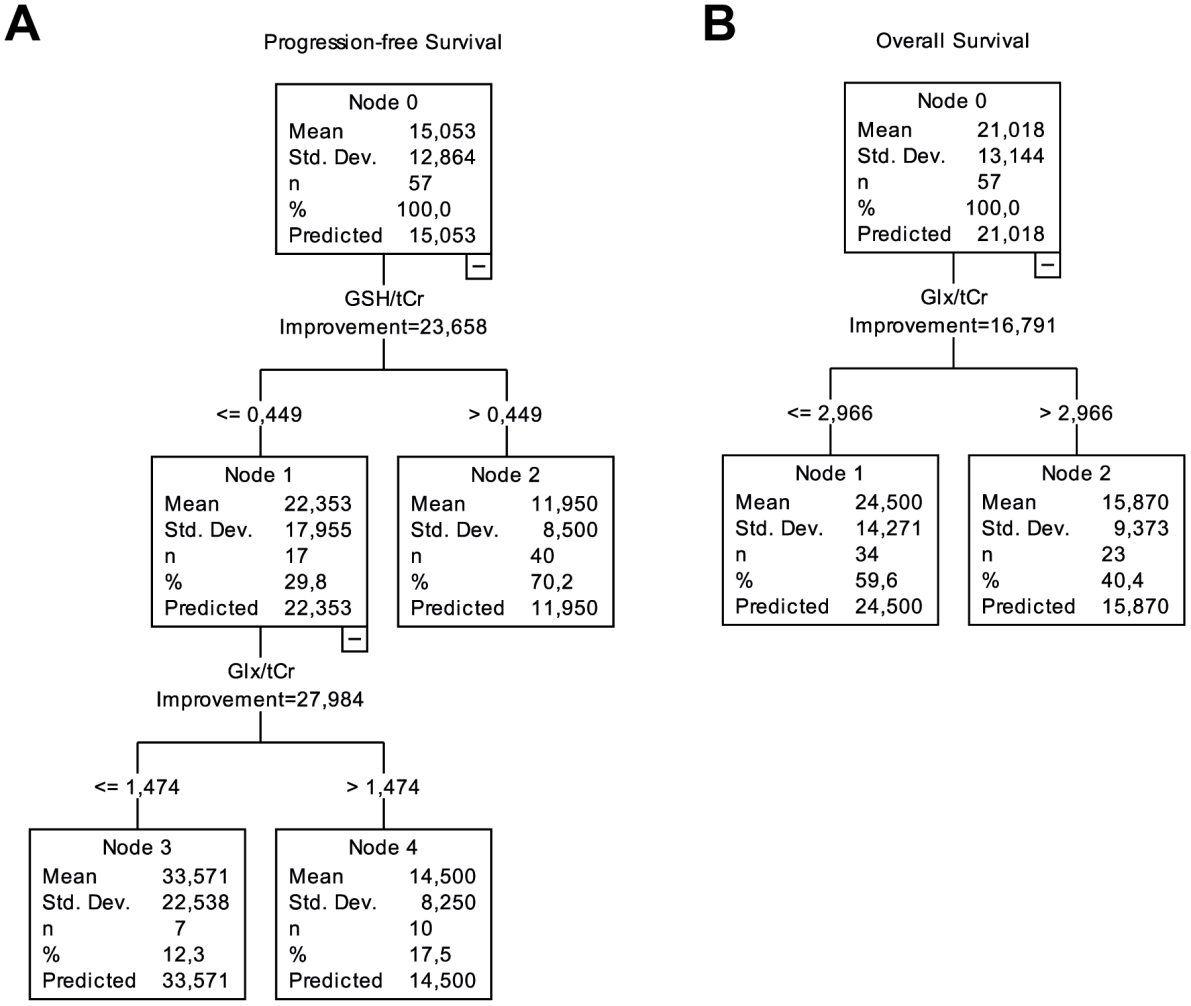
CART analysis for PFS and OS performed with clinical, imaging, and metabolic data from IDH-wt, TERTp-mut gliomas. **(A)** While patients with higher GSH/tCr (>0.449) had the worst PFS, the patients with lower GSH/tCr (≤0.449) and lower Glx/tCr (≤1.474) had a better PFS. **(B)** Higher Glx/tCr (>2.966) was associated with shorter OS.

CART analysis of “IDH-wt, TERTp-mut diffuse gliomas” indicated that a high GSH/tCr (>0.449), and a high Glx/tCr (>1.474) were associated with a significantly shorter PFS (Figure 4A). Additionally, high Glx/tCr (>2.966) was associated with a significantly shorter OS (Figure 4B). The sensitivity, specificity, and accuracy results along with the classification model, feature selection method, and selected metabolites for classification according to high PFS or OS are presented in Table 4. The models using MRS markers had better performance after oversampling than both the baseline models using only clinical features and the models that incorporated clinical features along with the MRS markers. The highest classification accuracy based on 12 months OS was 70.37%, but the specificity was lower (41.53%), since 67% of the patients had a longer than 12 months OS. Oversampling assisted models to learn better about the patients with longer OS, and following SMOTE, the specificity increased to 76.36% (accuracy = 81.71%, sensitivity = 87.05%). The most useful features for the classification of low and high OS were the extent of Glx/tCr, tCho/tCr, tNAA/tCr and (mIns+Glyc)/tCr. Similarly, for PFS based classification, the accuracy of the machine learning models for identifying the patients having longer than 6 months PFS was not very high (best accuracy = 63.93%, sensitivity = 68.41%, specificity = 54.22% using the MRS markers models). SMOTE also improved the PFS classification results for the minority class, resulting in an accuracy of 77.41% (sensitivity = 75.47%, specificity = 79.48%). Lac/tCr, tNAA/tCr, Glx/tCr and (mIns+Glyc)/tCr were used in this prediction.

**Table 4:** The performance results of machine learning models with the highest accuracies for different feature selection approaches with or without SMOTE oversampling.

Classification	Feature selection method	Selected features	Classification model	*n*_original_ (shorter vs. longer survival)	Original Acc/Sens/Spec (%)	*n*_oversampled_ (shorter vs. longer survival)	SMOTE Acc/Sens/Spec (%)
12 months OS	–	Age, Extent of resection, Contrast enhancement, Necrosis	Fine KNN	19 vs. 38	62.58/73.02/41.68	38 vs. 38	65.55/62.08/69.03
12 months OS	SFFS	Glx/tCr, tCho/tCr, tNAA/tCr, (mIns+Glyc)/tCr	Fine Gaussian SVM	19 vs. 38	70.37/84.81/41.53	38 vs. 38	81.71/87.05/76.36
12 months OS	Lasso	Necrosis, mIns/tCr, tNAA/tCr	Fine Gaussion SVM	19 vs. 38	67.16/88.05/25.37	38 vs. 38	75.16/65.21/85.11
6 months PFS	–	Age, extent of resection, contrast enhancement, necrosis	Fine KNN	18 vs. 39	61.95/75.67/32.22	39 vs. 39	72.82/67.38/78.26
6 months PFS	RFE	Lac/tCr, tNAA/tCr, (mIns+Glyc)/tCr, Glx/tCr	Subspace KNN	18 vs. 39	63.93/68.41/54.22	39 vs. 39	77.41/75.47/79.48
6 months PFS	SFFS	Necrosis, EOR, tNAA/tCr, (mIns+Glyc)/tCr	Fine KNN	18 vs. 39	65.19/29.44/81.69	39 vs. 39	76.39/64.72/88.05

The machine learning results for predicting short PFS and OS using the combinations of all different models and three different feature selection algorithms with or without oversampling are provided at Supplementary Tables S1 and S2, respectively. Median AUC for all the models when Lasso was employed as a feature selection algorithm to predict short OS was 0.82 [0.5–1]. On the other hand, the median AUC values were 0.84 [0.52–1] and 0.83 [0.53–1] when SFFS and RFE were employed for predicting short OS, respectively. For the oversampled dataset, the median AUC value was 0.85 [0.67–1] when Lasso was used as a feature selection algorithm. The median AUC values were 0.86 [0.62–1] and 0.85 [0.65–1] when SFFS and RFE were employed for predicting short OS, respectively.

The median AUC for all models, when utilizing Lasso as the feature selection algorithm to predict short PFS, was 0.84 [0.45–1]. On the other hand, when SFFS and RFE were employed to predict short PFS, the median AUC values were 0.88 [0.49–1] and 0.83 [0.54–1], respectively. When examining the oversampled dataset, the median AUC value for predicting short PFS was 0.91 [0.56–1] when utilizing Lasso as the feature selection algorithm. Similarly, when applying SFFS and RFE to predict short overall survival (OS) with the oversampled dataset, the median AUC values were 0.89 [0.57–1] and 0.92 [0.57–1], respectively.

## Discussion

Molecular subsets of gliomas are useful guidelines for understanding tumor biology, treatment response, and patient survival (Eckel-Passow et al., 2015). When combined with age group, anatomical localization, and histopathological findings, molecular markers provide significant clues for defining the tumor type of an adult diffuse glioma. The largest group among adult diffuse gliomas is glioblastoma, which is highly heterogeneous in its presentation, radiological and growth characteristics, treatment response, and survival. As a part of this spectrum, glioblastoma can also present with radiological features of a lower-grade diffuse glioma; nevertheless, they have a more aggressive clinical course than other lower-grade counterparts. Therefore, the 2021 WHO CNS tumor classification suggested diagnosing lower-grade astrocytic diffuse gliomas with specific molecular features as (glioblastoma, IDHwt) (Louis et al., 2021). This study has shown significant metabolic correlates of the patient survival in IDH-wt, TERTp-mut glioblastomas. These metabolic differences, as identified by short-TE ^1^H-MRS with machine learning algorithms, could help with predicting patient survival, independent of conventional MRI features such as contrast enhancement or necrosis. Although previous studies have investigated the relationship between anatomical, diffusion, and perfusion MR imaging markers in TERTp-mutant gliomas, metabolic imaging correlates of TERTp mutations have not been explored in detail (Yamashita et al., 2019; Park et al., 2020). In a previous study, “IDH-wt, TERTp-mut” diffuse gliomas were reported to have higher Glu and GSH levels than “IDH-mut, TERTp-wt,” “IDH-mut, TERTp-mut,” and “IDH-wt, TERT-wt” diffuse gliomas (Ozturk-Isik et al., 2019). Moreover, in the same study, TERTp-mut gliomas were shown to have higher Cr, Glu, and Glx levels than TERTp-wt gliomas. Our results indicated that higher ratios of GSH/tCr, tCho/tCr, and Glx/tCr were correlated with worse patient survival in IDH-wt, TERTp-mut glioblastomas.

GSH is an important cellular antioxidant in the brain and plays a significant role in protecting cells against free radical damage and cytotoxicity of chemotherapy (Backos et al., 2012). Several studies have reported a correlation between elevated GSH levels and TERTp mutation (Borrás et al., 2004; Viswanath et al., 2021; Minami et al., 2022). Our results also indicated that higher GSH/tCr ratios were associated with the presence of necrosis and shorter PFS in “IDH-wt TERTp-mut” gliomas. An increase in choline, which is an important intermediate in membrane phospholipid metabolism, is widely accepted as an indicator of cell proliferation in MRS (Nelson, 2001). In this current analysis, the presence of necrosis, which by itself is a determinant of IDH-wt glioblastoma, was associated with higher tCho/tCr levels in “IDH-wt TERTp-mut diffuse gliomas.” The excitatory neurotransmitters Glu and Gln levels were correlated with the presence of contrast enhancement in glioblastomas (Yan et al., 2019; Hangel et al., 2020). Similarly, in this study, their combined concentration (Glx/tCr) levels were associated with the presence of contrast enhancement, necrosis, shorter OS, and shorter PFS. Lac, a metabolic intermediate of anaerobic glycolysis (Horská and Barker, 2010), has been associated with tumor aggressiveness in gliomas (Vlassenko et al., 2015). Similarly, we observed higher Lac/tCr levels in tumors showing contrast enhancement or necrosis. Our findings indicated that high Glx and GSH to tCr ratios might be indicative of a more aggressive onco-metabolic profile and shorter survival in IDH-wt, TERTp-mut gliomas.

Several studies have indicated that WHO 2016 grade-2/3 and WHO 2016 grade-4 IDH-wt, TERTp-mut gliomas had a comparable OS (Reuss et al., 2015; Tesileanu et al., 2020). In contrast, other authors have reported longer survival in lower-grade astrocytic gliomas with molecular features of glioblastoma than in their grade-4 counterparts (Danyeli et al., 2019; Berzero et al., 2021). In our study, the OS difference between WHO 2016 grade-2/3 and WHO 2016 grade-4 tumors did not reach a statistically significant level, which supports the 2021 WHO CNS tumor classification criteria for IDH-wt gliomas.

Our survival analysis results indicated that the EOR was not significantly correlated with neither PFS nor OS of glioblastoma, IDH-wildtype, WHO grade-4 in this patient cohort. On the contrary, a recent systematic review has indicated that the gross total resection was associated with improved OS and PFS compared with subtotal resection or biopsy for glioblastoma, IDH-WT, WHO grade 4 (WHO 2021) (Jusue-Torres et al., 2023). This discrepancy might have resulted from the limited number of patients in our study and the clinical and methodological heterogeneity among the studies included in the recent review paper.

CART analyses revealed the importance of GSH/tCr, and Glx/tCr levels in terms of PFS and the importance of Glx/tCr level in terms of OS, which were in good agreement with our results of Kaplan–Meier and Cox regression analyses. Several supervised machine learning models and feature selection methods have been employed to classify IDH-wt, TERTp-mut gliomas based on their PFS and OS. According to our machine learning results, the data imbalance resulted in a lower accuracy for the minority class, and oversampling improved the classification results. Despite the small sample size, machine learning models provided promising results for survival prediction in IDH-wt, TERTp-mut gliomas using metabolic markers.

There was a discrepancy between the univariate Cox regression and machine learning analyses in terms of which metabolites were important. This difference might result from the univariate Cox regression analysis evaluating features independently rather than evaluating their combinational effects like in machine learning. Another reason for this discrepancy may be the algorithmic differences between the Cox regression analysis and different feature selection methods used in the machine learning methods. The algorithm used in Cox regression is primarily based on the partial likelihood estimation method, whereas SFFS is a feature selection approach that iteratively adds or removes features based on their impact on model performance, while RFE recursively removes features based on their importance rankings. On the other hand, LASSO inherently selects features by penalizing high coefficients using L1 norm.

This study had several limitations. First, our analysis was limited by its retrospective nature and small sample size, and our data were obtained from a single center. The results could be confirmed in a prospective multicenter study with a larger cohort. Additionally, spectral editing techniques might be used to increase the sensitivity of detecting small and overlapping metabolites (Choi and Lee, 2013). However, these techniques require longer imaging times. Although GSH is difficult to reliably quantify due to the overlapping resonances of other metabolites within the human brain, such as mIns, Gln and Glu (Govindaraju et al., 2001), a recent study has shown that non-editing techniques, including short echo time PRESS, could reproducibly quantify GSH (Wijtenburg et al., 2019). As a result, short-TE MRS was preferred in this study, which is widely available in the clinics and provides diagnostic information about multiple spectral peaks. Moreover, we did not have unsuppressed water available for our dataset and could not perform absolute metabolite quantification. Furthermore, several studies have suggested that TERTp mutation alone may be insufficient, and the presence of EGFR amplification and 7+/10- pattern might also affect the prognosis of IDH-wt, TERTp-mut WHO 2016 grade-2/3 gliomas (Wijnenga et al., 2017; Stichel et al., 2018). This genetic information was not available for all of our patients, and future studies are needed to explore the metabolic markers in IDH-wt, TERTp-mut gliomas in light of further genetic markers.

## Conclusion

In conclusion, Glx and GSH have been identified as important metabolic correlates of patient survival among “IDH-wt, TERTp-mut diffuse gliomas” using single-voxel ^1^H-MRS on a clinical 3 T MRI scanner.

## Data availability statement

The datasets presented in this article are not readily available because all data was obtained upon retrospective review of patient medical records and are not publicly available. Requests to access the datasets should be directed to esin.ozturk@boun.edu.tr.

## Ethics statement

The studies involving human participants were reviewed and approved by Institutional Review Board of Acibadem Mehmet Ali Aydinlar University School of Medicine (ATADEK 2012-292). The patients/participants provided their written informed consent to participate in this study.

## Author contributions

BS-B performed the analysis and interpretation of the data and drafting of the manuscript. AED performed the pathological analysis and critical revision. MCY performed the genetic testing and critical revision. FKA performed the extent of surgical resection evaluations, manuscript editing, and critical revision. KÖ and MNP performed the patient recruitment, clinical evaluation, and critical revision. KÖ and EO-I performed the study conception and design, manuscript editing, and critical revision. AD performed the radiological data acquisition and evaluation, and critical revision. All authors contributed to the article and approved the submitted version.

## Funding

This study was supported by the Scientific and Technological Research Council of Turkey (TUBITAK) 1003 grant (216S432).

## Conflict of interest

The authors declare that the research was conducted in the absence of any commercial or financial relationships that could be construed as a potential conflict of interest.

## Publisher’s note

All claims expressed in this article are solely those of the authors and do not necessarily represent those of their affiliated organizations, or those of the publisher, the editors and the reviewers. Any product that may be evaluated in this article, or claim that may be made by its manufacturer, is not guaranteed or endorsed by the publisher.
