# Supplementary material for: Magnetic resonance spectroscopic correlates of progression free and overall survival in “glioblastoma, IDH-wildtype, WHO grade-4”
Source: Front Neurosci. 2023 Jun 29;17:1149292. doi: 10.3389/fnins.2023.1149292 (PMC10339315; doi:10.3389/fnins.2023.1149292)
Supplement: Supplementary file 1 [file Data_Sheet_1.docx]

**Supplementary Material**

**
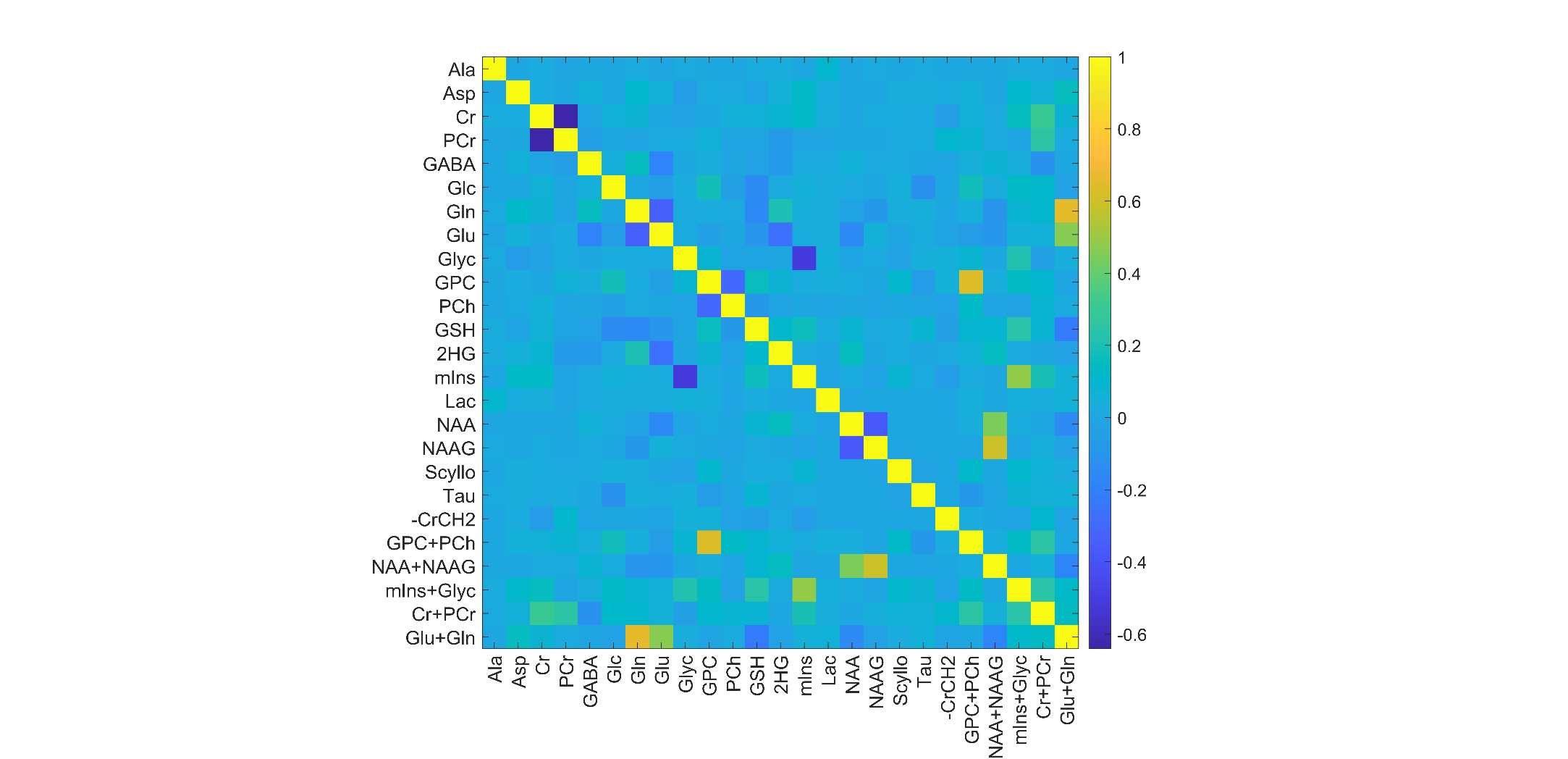
**

**Figure S1** Mean correlation matrix output of the LCModel averaged over all patients


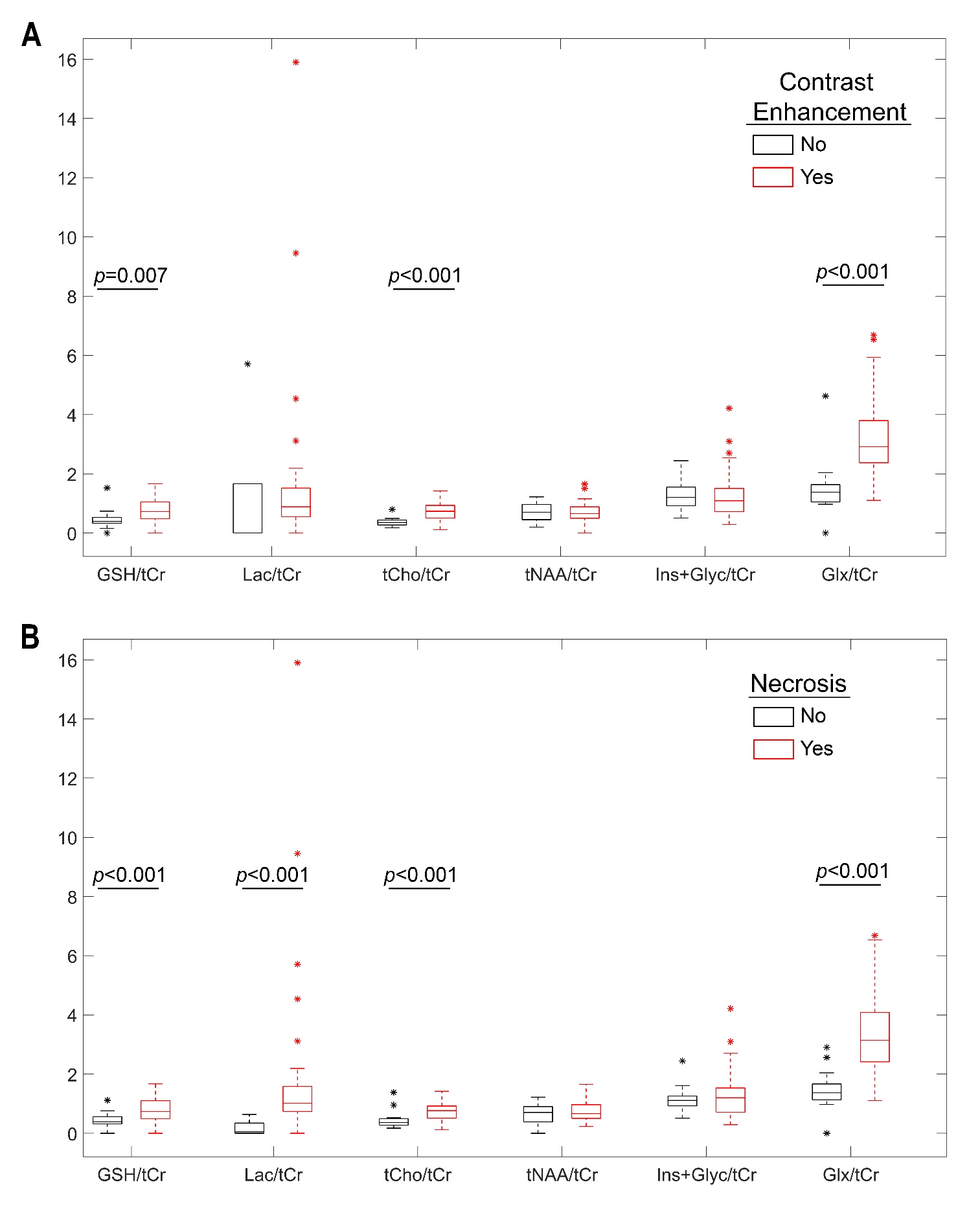


**Figure S2.** Boxplots of metabolite/total creatinine (tCr) ratios of **(A)** contrast-enhanced/non-contrast-enhanced tumors and **(B)** tumors with/without necrosis in patients with IDH-wt, TERTp-mut glioma.


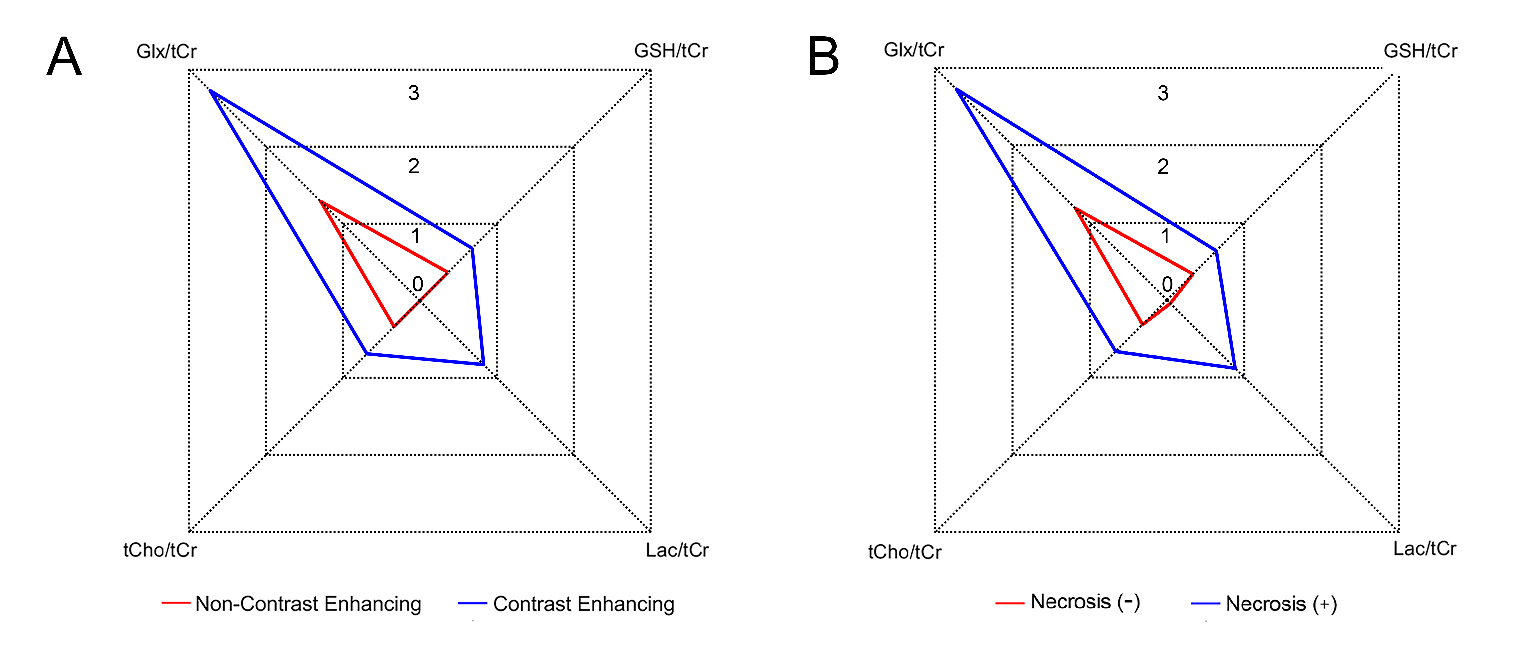
**Figure S3** Contrast-enhanced tumors had GSH/tCr, tCho/tCr, and Glx/tCr levels and lower mIns/tCr levels than non-contrast-enhanced tumors (A). Tumors with necrosis had higher GSH/tCr, Lac/tCr, tCho/tCr, and Glx/tCr levels and lower mIns/tCr levels than the other tumors (B).

Table S1. The machine learning results for the combinations of all different models and three different feature selection algorithms developed for PFS.

| Model | Original dataset | | | Oversampled dataset using SMOTE | | |
| --- | --- | --- | --- | --- | --- | --- |
|  | Lasso | SFFS | RFE | Lasso | SFFS | RFE |
| Fine Tree | Acc: 0.55 [0.46-0.61]  Sen: 0.67 [0.56-0.74]  Spe: 0.28 [0.17-0.44]  AUC: 0.78 [0.72-0.85] | Acc: 0.57 [0.44-0.61]  Sen: 0.71 [0.59-0.82]  Spe: 0.22 [0.11-0.44]  AUC: 0.84 [0.77-0.89] | Acc: 0.54 [0.46-0.60]  Sen: 0.69 [0.51-0.77]  Spe: 0.22 [0.17-0.44]  AUC: 0.84 [0.72-0.94] | Acc: 0.63 [0.56-0.72]  Sen: 0.59 [0.41-0.69]  Spe: 0.69 [0.53-0.79]  AUC: 0.91 [0.86-0.95] | Acc: 0.59 [0.51-0.62]  Sen: 0.59 [0.49-0.72]  Spe: 0.56 [0.49-0.62]  AUC: 0.89 [0.84-0.94] | Acc: 0.66 [0.62-0.72]  Sen: 0.69 [0.59-0.82]  Spe: 0.62 [0.62-0.69]  AUC: 0.92 [0.90-0.97] |
| Medium Tree | Acc: 0.56 [0.47-0.65]  Sen: 0.65 [0.62-0.74]  Spe: 0.31 [0.11-0.44]  AUC: 0.80 [0.73-0.90] | Acc: 0.54 [0.49-0.65]  Sen: 0.65 [0.62-0.74]  Spe: 0.31 [0.11-0.44]  AUC: 0.83 [0.73-0.89] | Acc: 0.54 [0.47-0.60]  Sen: 0.64 [0.59-0.72]  Spe: 0.28 [0.17-0.33]  AUC: 0.83 [0.71-0.93] | Acc: 0.65 [0.54-0.69]  Sen: 0.58 [0.51-0.64]  Spe: 0.28 [0.17-0.33]  AUC: 0.91 [0.88-0.96] | Acc: 0.56 [0.47-0.64]  Sen: 0.59 [0.41-0.72]  Spe: 0.53 [0.49-0.67]  AUC: 0.89 [0.86-0.93] | Acc: 0.67 [0.65-0.73]  Sen: 0.71 [0.64-0.82]  Spe: 0.64 [0.62-0.72]  AUC: 0.94 [0.89-0.96] |
| Coarse Tree | Acc: 0.58 [0.53-0.61]  Sen: 0.73 [0.67-0.79]  Spe: 0.28 [0.17-0.33]  AUC: 0.74 [0.70-0.83] | Acc: 0.56 [0.47-0.61]  Sen: 0.73 [0.64-0.80]  Spe: 0.22 [0.10-0.28]  AUC: 0.81 [0.76-0.87] | Acc: 0.56 [0.44-0.58]  Sen: 0.71 [0.62-0.79]  Spe: 0.22 [0.10-0.28]  AUC: 0.78 [0.73-0.85] | Acc: 0.56 [0.50-0.60]  Sen: 0.55 [0.49-0.62]  Spe: 0.56 [0.51-0.59]  AUC: 0.77 [0.74-0.82] | Acc: 0.58 [0.55-0.64]  Sen: 0.55 [0.51-0.64]  Spe: 0.62 [0.54-0.69]  AUC: 0.78 [0.74-0.83] | Acc: 0.55 [0.51-0.61]  Sen: 0.55 [0.49-0.74]  Spe: 0.51 [0.41-0.59]  AUC: 0.81 [0.72-0.82] |
| Linear Discriminant | Acc: 0.66 [0.61-0.68]  Sen: 0.82 [0.77-0.85]  Spe: 0.33 [0.22-0.33]  AUC: 0.68 [0.65-0.72] | Acc: 0.49 [0.42-0.56]  Sen: 0.62 [0.56-0.72]  Spe: 0.22 [0.10-0.33]  AUC: 0.79 [0.74-0.81] | Acc: 0.57 [0.51-0.68]  Sen: 0.69 [0.62-0.79]  Spe: 0.33 [0.11-0.44]  AUC: 0.69 [0.64-0.76] | Acc: 0.60 [0.56-0.68]  Sen: 0.54 [0.49-0.61]  Spe: 0.33 [0.11-0.44]  AUC: 0.78 [0.76-0.81] | Acc: 0.58 [0.53-0.59]  Sen: 0.37 [0.31-0.39]  Spe: 0.77 [0.74-0.79]  AUC: 0.76 [0.71-0.78] | Acc: 0.56 [0.51-0.59]  Sen: 0.33 [0.23-0.41]  Spe: 0.79 [0.74-0.82]  AUC: 0.74 [0.71-0.78] |
| Quadratic Discriminant | Acc: 0.65 [0.63-0.68]  Sen: 0.82 [0.79-0.84]  Spe: 0.28 [0.22-0.33]  AUC: 0.77 [0.71-0.79] | Acc: 0.52 [0.44-0.56]  Sen: 0.68 [0.54-0.74]  Spe: 0.17 [0.11-0.28]  AUC: 0.77 [0.73-0.80] | Acc: 0.58 [0.54-0.63]  Sen: 0.72 [0.64-0.77]  Spe: 0.33 [0.17-0.39]  AUC: 0.69 [0.63-0.75] | Acc: 0.62 [0.56-0.68]  Sen: 0.54 [0.44-0.62]  Spe: 0.69 [0.62-0.77]  AUC: 0.80 [0.77-0.82] | Acc: 0.56 [0.49-0.59]  Sen: 0.33 [0.28-0.41]  Spe: 0.77 [0.69-0.77]  AUC: 0.77 [0.73-0.80] | Acc: 0.55 [0.50-0.56]  Sen: 0.33 [0.26-0.36]  Spe: 0.77 [0.72-0.82]  AUC: 0.74 [0.71-0.78] |
| Logistic Regression | Acc: 0.66 [0.58-0.68]  Sen: 0.87 [0.80-0.92]  Spe: 0.17 [0.10-0.22]  AUC: 0.53 [0.45-0.57] | Acc: 0.60 [0.56-0.67]  Sen: 0.82 [0.79-0.87]  Spe: 0.14 [0-0.22]  AUC: 0.56 [0.49-0.58] | Acc: 0.63 [0.60-0.67]  Sen: 0.85 [0.79-0.90]  Spe: 0.17 [0.17-0.22]  AUC: 0.57 [0.54-0.58] | Acc: 0.56 [0.53-0.58]  Sen: 0.56 [0.53-0.62]  Spe: 0.54 [0.51-0.59]  AUC: 0.58 [0.56-0.61] | Acc: 0.56 [0.50-0.59]  Sen: 0.55 [0.49-0.56]  Spe: 0.59 [0.51-0.61]  AUC: 0.59 [0.57-0.60] | Acc: 0.59 [0.54-0.60]  Sen: 0.56 [0.51-0.59]  Spe: 0.59 [0.56-0.64]  AUC: 0.57 [0.53-0.60] |
| Quadratic SVM | Acc: 0.63 [0.58-0.65]  Sen: 0.88 [0.84-0.92]  Spe: 0.1 [0-0.11]  AUC: 0.99 [0.99-1] | Acc: 0.60 [0.58-0.68]  Sen: 0.86 [0.82-1]  Spe: 0 [0-0.1]  AUC: 0.99 [0.99-1] | Acc: 0.62 [0.56-0.70]  Sen: 0.81 [0.77-0.90]  Spe: 0.17 [0.11-0.28]  AUC: 0.99 [0.97-1] | Acc: 0.70 [0.64-0.73]  Sen: 0.60 [0.54-0.67]  Spe: 0.69 [0.62-0.77]  AUC: 0.88 [0.86-0.89] | Acc: 0.69 [0.67-0.71]  Sen: 0.62 [0.67-0.74]  Spe: 0.72 [0.69-0.74]  AUC: 0.89 [0.86-0.91] | Acc: 0.71 [0.67-0.78]  Sen: 0.67 [0.62-0.74]  Spe: 0.74 [0.69-0.82]  AUC: 0.87 [0.85-0.89] |
| Cubic SVM | Acc: 0.60 [0.58-0.61]  Sen: 0.72 [0.67-0.74]  Spe: 0.22 [0.22-0.39]  AUC: 0.96 [0.86-1] | Acc: 0.58 [0.54-0.65]  Sen: 0.71 [0.62-0.74]  Spe: 0.36 [0.22-0.44]  AUC: 0.94 [0.92-0.99] | Acc: 0.63 [0.53-0.65]  Sen: 0.76 [0.64-0.82]  Spe: 0.36 [0.22-0.44]  AUC: 0.92 [0.87-0.99] | Acc: 0.72 [0.65-0.74]  Sen: 0.66 [0.56-0.69]  Spe: 0.77 [0.69-0.80]  AUC: 0.98 [0.96-1] | Acc: 0.64 [0.60-0.68]  Sen: 0.59 [0.46-0.64]  Spe: 0.69 [0.64-0.74]  AUC: 0.95 [0.92-0.99] | Acc: 0.68 [0.59-0.74]  Sen: 0.64 [0.51-0.72]  Spe: 0.72 [0.56-0.82]  AUC: 0.97 [0.95-0.99] |
| Fine Gaussian SVM | Acc: 0.68 [0.67-0.68]  Sen: 1 [0.97-1]  Spe: 0 [0-0]  AUC: 0.99 [0.98-0.99] | Acc: 0.68 [0.67-0.68]  Sen: 1 [0.97-1]  Spe: 0 [0-0]  AUC: 0.99 [0.99-0.99] | Acc: 0.68 [0.68-0.68]  Sen: 1 [1-1]  Spe: 0 [0-0]  AUC: 0.99 [0.94-0.99] | Acc: 0.72 [0.70-0.75]  Sen: 0.82 [0.74-0.87]  Spe: 0.67 [0.59-0.69]  AUC: 0.99 [0.99-1] | Acc: 0.67 [0.65-0.69]  Sen: 0.79 [0.77-0.85]  Spe: 0.54 [0.49-0.59]  AUC: 0.98 [0.98-1] | Acc: 0.68 [0.65-0.73]  Sen: 0.72 [0.69-0.79]  Spe: 0.64 [0.62-0.69]  AUC: 0.99 [0.99-1] |
| Fine KNN | Acc: 0.58 [0.51-0.58]  Sen: 0.65 [0.56-0.69]  Spe: 0.44 [0.33-0.44]  AUC: 0.96 [0.94-0.98] | Acc: 0.54 [0.51-0.58]  Sen: 0.67 [0.64-0.69]  Spe: 0.28 [0.16-0.33]  AUC: 0.95 [0.93-1] | Acc: 0.61 [0.58-0.68]  Sen: 0.72 [0.67-0.77]  Spe: 0.39 [0.28-0.50]  AUC: 0.97 [0.96-0.99] | Acc: 0.72 [0.68-0.74]  Sen: 0.58 [0.54-0.62]  Spe: 0.86 [0.77-0.90]  AUC: 0.96 [0.96-0.99] | Acc: 0.67 [0.65-0.69]  Sen: 0.55 [0.49-0.66]  Spe: 0.87 [0.79-0.90]  AUC: 0.96 [0.94-1] | Acc: 0.79 [0.73-0.82]  Sen: 0.69 [0.62-0.72]  Spe: 0.90 [0.85-0.92]  AUC: 0.98 [0.95-1] |
| Medium KNN | Acc: 0.59 [0.56-0.61]  Sen: 0.85 [0.79-0.87]  Spe: 0.1 [0-0.1]  AUC: 0.98 [0.96-1] | Acc: 0.62 [0.60-0.67]  Sen: 0.87 [0.85-0.95]  Spe: 0.1 [0.1-0.17]  AUC: 0.98 [0.96-1] | Acc: 0.63 [0.60-0.67]  Sen: 0.85 [0.79-0.87]  Spe: 0.17 [0.11-0.28]  AUC: 0.98 [0.96-1] | Acc: 0.63 [0.59-0.68]  Sen: 0.50 [0.49-0.59]  Spe: 0.76 [0.67-0.85]  AUC: 0.98 [0.96-1] | Acc: 0.65 [0.60-0.69]  Sen: 0.54 [0.46-0.59]  Spe: 0.72 [0.69-0.82]  AUC: 0.98 [0.96-1] | Acc: 0.67 [0.64-0.72]  Sen: 0.56 [0.51-0.64]  Spe: 0.78 [0.69-0.87]  AUC: 0.98 [0.96-1] |
| Bagged Tree | Acc: 0.64 [0.51-0.68]  Sen: 0.77 [0.72-0.90]  Spe: 0.31 [0.1-0.44]  AUC: 0.99 [0.96-1] | Acc: 0.55 [0.54-0.60]  Sen: 0.74 [0.69-0.77]  Spe: 0.22 [0.11-0.28]  AUC: 0.99 [0.95-1] | Acc: 0.60 [0.54-0.67]  Sen: 0.77 [0.72-0.82]  Spe: 0.22 [0.11-0.39]  AUC: 0.99 [0.96-1] | Acc: 0.69 [0.65-0.71]  Sen: 0.58 [0.54-0.67]  Spe: 0.76 [0.67-0.79]  AUC: 0.99 [0.98-1] | Acc: 0.63 [0.58-0.71]  Sen: 0.64 [0.59-0.72]  Spe: 0.68 [0.62-0.74]  AUC: 0.99 [0.96-1] | Acc: 0.74 [0.68-0.77]  Sen: 0.66 [0.59-0.72]  Spe: 0.82 [0.74-0.85]  AUC: 0.99 [0.98-1] |
| Subspace discriminant | Acc: 0.67 [0.61-0.70]  Sen: 0.91 [0.85-0.95]  Spe: 0.1 [0.1-0.17]  AUC: 0.67 [0.66-0.69] | Acc: 0.60 [0.58-0.61]  Sen: 0.87 [0.85-0.90]  Spe: 0 [0-0.1]  AUC: 0.71 [0.69-0.72] | Acc: 0.65 [0.61-0.67]  Sen: 0.89 [0.82-0.92]  Spe: 0.1 [0.1-0.17]  AUC: 0.68 [0.67-0.70] | Acc: 0.56 [0.51-0.60]  Sen: 0.54 [0.49-0.59]  Spe: 0.56 [0.51-0.64]  AUC: 0.68 [0.66-0.70] | Acc: 0.55 [0.51-0.59]  Sen: 0.54 [0.49-0.59]  Spe: 0.56 [0.51-0.64]  AUC: 0.68 [0.68-0.70] | Acc: 0.59 [0.58-0.61]  Sen: 0.58 [0.56-0.61]  Spe: 0.61 [0.56-0.64]  AUC: 0.69 [0.68-0.70] |
| Subspace KNN | Acc: 0.61 [0.58-0.67]  Sen: 0.77 [0.74-0.85]  Spe: 0.28 [0.22-0.33]  AUC: 0.84 [0.81-0.86] | Acc: 0.63 [0.60-0.70]  Sen: 0.72 [0.69-0.82]  Spe: 0.44 [0.33-0.55]  AUC: 0.88 [0.83-0.89] | Acc: 0.64 [0.58-0.68]  Sen: 0.79 [0.74-0.82]  Spe: 0.31 [0.17-0.44]  AUC: 0.85 [0.80-0.86] | Acc: 0.78 [0.76-0.80]  Sen: 0.64 [0.59-0.67]  Spe: 0.91 [0.87-0.92]  AUC: 0.99 [0.98-1] | Acc: 0.77 [0.74-0.80]  Sen: 0.65 [0.62-0.69]  Spe: 0.90 [0.85-0.92]  AUC: 0.99 [0.96-1] | Acc: 0.69 [0.67-0.73]  Sen: 0.60 [0.54-0.64]  Spe: 0.79 [0.74-0.82]  AUC: 0.98 [0.98-1] |
| RUSBoosted Trees | Acc: 0.51 [0.44-0.56]  Sen: 0.54 [0.44-0.61]  Spe: 0.44 [0.28-0.61]  AUC: 1 [0.95-1] | Acc: 0.54 [0.44-0.67]  Sen: 0.59 [0.49-0.69]  Spe: 0.50 [0.28-0.61]  AUC: 0.99 [0.95-1] | Acc: 0.53 [0.46-0.54]  Sen: 0.54 [0.49-0.59]  Spe: 0.44 [0.39-0.56]  AUC: 0.79 [0.79-1] | Acc: 0.52 [0.48-0.59]  Sen: 0.1 [0.1-0.23]  Spe: 0.97 [0.85-1]  AUC: 0.94 [0.79-0.99] | Acc: 0.52 [0.50-0.54]  Sen: 0 [0-0.1]  Spe: 1 [0.95-1]  AUC: 0.86 [0.79-0.98] | Acc: 0.50 [0.49-0.56]  Sen: 0.1 [0-0.1]  Spe: 0.99 [0.95-1]  AUC: 0.79 [0.79-0.96] |

Table S2. The machine learning results for the combinations of all different models and three different feature selection algorithms developed for OS.

| Model | Original dataset | | | Oversampled dataset using SMOTE | | |
| --- | --- | --- | --- | --- | --- | --- |
|  | Lasso | SFFS | RFE | Lasso | SFFS | RFE |
| Fine Tree | Acc: 0.64 [0.54-0.72]  Sen: 0.76 [0.68-0.82]  Spe: 0.40 [0.21-0.53]  AUC: 0.88 [0.86-0.91] | Acc: 0.57 [0.46-0.60]  Sen: 0.70 [0.53-0.76]  Spe: 0.32 [0.21-0.37]  AUC: 0.87 [0.80-0.92] | Acc: 0.57 [0.53-0.63]  Sen: 0.71 [0.63-0.84]  Spe: 0.32 [0.21-0.37]  AUC: 0.88 [0.83-92] | Acc: 0.72 [0.68-0.78]  Sen: 0.76 [0.68-0.79]  Spe: 0.68 [0.61-0.69]  AUC: 0.90 [0.85-0.92] | Acc: 0.66 [0.62-0.70]  Sen: 0.68 [0.58-0.82]  Spe: 0.62 [0.53-0.71]  AUC: 0.91 [0.83-0.93] | Acc: 0.66 [0.59-0.71]  Sen: 0.70 [0.61-0.74]  Spe: 0.65 [0.50-0.74]  AUC: 0.91 [0.86-0.94] |
| Medium Tree | Acc: 0.62 [0.58-0.65]  Sen: 0.74 [0.71-0.82]  Spe: 0.37 [0.26-0.47]  AUC: 0.87 [0.84-0.90] | Acc: 0.58 [0.49-0.61]  Sen: 0.70 [0.58-0.76]  Spe: 0.32 [0.21-0.37]  AUC: 0.87 [0.82-0.91] | Acc: 0.59 [0.53-0.65]  Sen: 0.72 [0.68-0.79]  Spe: 0.26 [0.16-0.47]  AUC: 0.85 [0.82-0.92] | Acc: 0.73 [0.68-0.76]  Sen: 0.74 [0.66-0.79]  Spe: 0.74 [0.63-0.84]  AUC: 0.91 [0.87-0.93] | Acc: 0.66 [0.59-0.71]  Sen: 0.67 [0.55-0.74]  Spe: 0.63 [0.55-0.68]  AUC: 0.90 [0.87-0.92] | Acc: 0.64 [0.59-0.70]  Sen: 0.68 [0.58-0.79]  Spe: 0.61 [0.47-0.71]  AUC: 0.91 [0.84-0.94] |
| Coarse Tree | Acc: 0.64 [0.60-0.68]  Sen: 0.67 [0.61-0.71]  Spe: 0.58 [0.47-0.68]  AUC: 0.81 [0.79-0.84] | Acc: 0.63 [0.49-0.68]  Sen: 0.74 [0.63-0.84]  Spe: 0.37 [0.21-0.47]  AUC: 0.83 [0.81-0.84] | Acc: 0.60 [0.56-0.68]  Sen: 0.64 [0.58-0.68]  Spe: 0.53 [0.37-0.63]  AUC: 0.80 [0.77-0.81] | Acc: 0.75 [0.70-0.78]  Sen: 0.68 [0.58-0.71]  Spe: 0.83 [0.76-0.87]  AUC: 0.85 [0.84-0.87] | Acc: 0.62 [0.57-0.67]  Sen: 0.58 [0.50-0.71]  Spe: 0.65 [0.55-0.76]  AUC: 0.83 [0.77-0.85] | Acc: 0.72 [0.67-0.76]  Sen: 0.63 [0.55-0.68]  Spe: 0.83 [0.74-0.89]  AUC: 0.83 [0.77-0.84] |
| Linear Discriminant | Acc: 0.56 [0.49-0.65]  Sen: 0.71 [0.66-0.82]  Spe: 0.21 [0.16-0.36]  AUC: 0.68 [0.65-0.71] | Acc: 0.57 [0.51-0.61]  Sen: 0.76 [0.68-0.84]  Spe: 0.15 [0.11-0.21]  AUC: 0.81 [0.77-0.87] | Acc: 0.59 [0.53-0.61]  Sen: 0.76 [0.68-0.84]  Spe: 0.26 [0.16-0.32]  AUC: 0.70 [0.67-0.76] | Acc: 0.62 [0.58-0.68]  Sen: 0.47 [0.39-0.50]  Spe: 0.76 [0.74-0.82]  AUC: 0.81 [0.79-0.85] | Acc: 0.64 [0.55-0.68]  Sen: 0.58 [0.47-0.66]  Spe: 0.70 [0.63-0.76]  AUC: 0.81 [0.79-0.85] | Acc: 0.64 [0.60-0.67]  Sen: 0.58 [0.50-0.61]  Spe: 0.71 [0.66-0.74]  AUC: 0.85 [0.82-0.89] |
| Quadratic Discriminant | Acc: 0.58 [0.49-0.61]  Sen: 0.75 [0.66-0.82]  Spe: 0.18 [0.11-0.26]  AUC: 0.69 [0.67-0.75] | Acc: 0.58 [0.56-0.63]  Sen: 0.79 [0.76-0.84]  Spe: 0.16 [0.11-0.26]  AUC: 0.79 | Acc: 0.58 [0.53-0.65]  Sen: 0.79 [0.71-0.84]  Spe: 0.16 [0.16-0.26]  AUC: 0.70 [0.67-0.78] | Acc: 0.59 [0.58-0.65]  Sen: 0.45 [0.37-0.54]  Spe: 0.76 [0.74-0.79]  AUC: 0.81 [0.79-0.782] | Acc: 0.64 [0.62-0.67]  Sen: 0.59 [0.53-0.68]  Spe: 0.70 [0.61-0.76]  AUC: 0.86 [0.83-0.88] | Acc: 0.62 [0.59-0.68]  Sen: 0.59 [0.53-0.68]  Spe: 0.70 [0.61-0.76]  AUC: 0.81 [0.79-0.88] |
| Logistic Regression | Acc: 0.60 [0.54-0.61]  Sen: 0.79 [0.74-0.79]  Spe: 0.21 [0.16-0.26]  AUC: 0.56 [0.50-0.58] | Acc: 0.60 [0.49-0.63]  Sen: 0.82 [0.74-0.84]  Spe: 0.16 [0-0.26]  AUC: 0.56 [0.52-0.60] | Acc: 0.61 [0.53-0.63]  Sen: 0.79 [0.74-0.82]  Spe: 0.21 [0.11-0.26]  AUC: 0.58 [0.53-0.61] | Acc: 0.64 [0.61-0.70]  Sen: 0.63 [0.61-0.68]  Spe: 0.64 [0.61-0.71]  AUC: 0.67 [0.65-0.68] | Acc: 0.62 [0.61-0.66]  Sen: 0.61 [0.58-0.63]  Spe: 0.65 [0.61-0.68]  AUC: 0.64 [0.62-0.67] | Acc: 0.65 [0.62-0.67]  Sen: 0.66 [0.58-0.68]  Spe: 0.64 [0.61-0.66]  AUC: 0.66 [0.64-0.68] |
| Quadratic SVM | Acc: 0.54 [0.44-0.56]  Sen: 0.66 [0.53-0.71]  Spe: 0.26 [0.16-0.37]  AUC: 0.82 [0.79-0.83] | Acc: 0.57 [0.53-0.61]  Sen: 0.71 [0.66-0.79]  Spe: 0.29 [0.21-0.37]  AUC: 0.84 [0.81-0.89] | Acc: 0.56 [0.54-0.65]  Sen: 0.68 [0.63-0.71]  Spe: 0.34 [0.26-0.53]  AUC: 0.83 [0.79-0.85] | Acc: 0.72 [0.66-0.74]  Sen: 0.61 [0.53-0.66]  Spe: 0.82 [0.79-0.87]  AUC: 0.87 [0.85-0.89] | Acc: 0.68 [0.66-0.72]  Sen: 0.61 [0.55-0.68]  Spe: 0.76 [0.74-0.79]  AUC: 0.87 [0.83-0.89] | Acc: 0.74 [0.71-0.76]  Sen: 0.64 [0.63-0.71]  Spe: 0.83 [0.76-0.87]  AUC: 0.88 [0.86-0.90] |
| Cubic SVM | Acc: 0.58 [0.51-0.63]  Sen: 0.68 [0.63-0.74]  Spe: 0.37 [0.21-0.47]  AUC: 0.94 [0.88-0.98] | Acc: 0.56 [0.49-0.61]  Sen: 0.68 [0.63-0.74]  Spe: 0.32 [0.21-0.42]  AUC: 0.94 [0.92-0.98] | Acc: 0.49 [0.46-0.60]  Sen: 0.55 [0.53-0.61]  Spe: 0.37 [0.26-0.58]  AUC: 0.91 [0.84-0.96] | Acc: 0.71 [0.67-0.75]  Sen: 0.63 [0.55-0.68]  Spe: 0.79 [0.74-0.87]  AUC: 0.95 [0.92-0.98] | Acc: 0.64 [0.59-0.68]  Sen: 0.56 [0.44-0.66]  Spe: 0.72 [0.66-0.74]  AUC: 0.96 [0.93-0.99] | Acc: 0.64 [0.59-0.68]  Sen: 0.56 [0.45-0.66]  Spe: 0.72 [0.66-0.74]  AUC: 0.94 [0.91-0.98] |
| Fine Gaussian SVM | Acc: 0.65 [0.65-0.67]  Sen: 0.77 [0.75-0.81]  Spe: 0.32 [0.25-0.34]  AUC: 0.95 [0.92-0.98] | Acc: 0.69 [0.67-0.72]  Sen: 0.84 [0.82-0.88]  Spe: 0.39 [0.35-0.47]  AUC: 0.95 [0.92-0.98] | Acc: 0.64 [0.60-0.66]  Sen: 0.78 [0.75-0.80]  Spe: 0.33 [0.25-0.39]  AUC: 0.95 [0.92-0.98] | Acc: 0.77 [0.76-0.80]  Sen: 0.87 [0.87-0.89]  Spe: 0.67 [0.63-0.71]  AUC: 0.99 [0.99-0.99] | Acc: 0.82 [0.79-0.84]  Sen: 0.87 [0.84-0.89]  Spe: 0.79 [0.74-0.79]  AUC: 0.99 [0.99-0.99] | Acc: 0.80 [0.76-0.83]  Sen: 0.91 [0.89-0.95]  Spe: 0.71 [0.63-0.74]  AUC: 0.99 [0.99-0.99] |
| Fine KNN | Acc: 0.54 [0.49-0.58]  Sen: 0.61 [0.58-0.66]  Spe: 0.42 [0.26-0.53]  AUC: 0.97 [0.93-0.99] | Acc: 0.61 [0.54-0.67]  Sen: 0.74 [0.66-0.79]  Spe: 0.40 [0.26-0.47]  AUC: 0.97 [0.93-1] | Acc: 0.53 [0.47-0.60]  Sen: 0.59 [0.50-0.68]  Spe: 0.42 [0.32-0.58]  AUC: 0.97 [0.93-1] | Acc: 0.70 [0.68-0.74]  Sen: 0.55 [0.50-0.61]  Spe: 0.87 [0.82-0.87]  AUC: 0.97 [0.96-1] | Acc: 0.74 [0.71-0.76]  Sen: 0.61 [0.58-0.66]  Spe: 0.87 [0.79-0.89]  AUC: 0.98 [0.96-0.99] | Acc: 0.70 [0.63-0.72]  Sen: 0.57 [0.45-0.58]  Spe: 0.87 [0.82-0.87]  AUC: 0.97 [0.93-0.99] |
| Boosted Trees | Acc: 0.33 [0.33-0.33]  Sen: 0 [0-0]  Spe: 1 [1-1]  AUC: 0.59 [0.59-0.59] | Acc: 0.33 [0.33-0.33]  Sen: 0 [0-0]  Spe: 1 [1-1]  AUC: 0.59 [0.59-0.59] | Acc: 0.33 [0.33-0.33]  Sen: 0 [0-0]  Spe: 1 [1-1]  AUC: 0.79 [0.79-0.79] | Acc: 0.5 [0.5-0.5]  Sen: 0 [0-0]  Spe: 1 [1-1]  AUC: 0.79 [0.79-0.79] | Acc: 0.5 [0.5-0.54]  Sen: 0 [0-0.1]  Spe: 1 [1-1]  AUC: 0.79 [0.79-0.79] | Acc: 0.5 [0.5-0.5]  Sen: 0 [0-0]  Spe: 1 [1-1]  AUC: 0.79 [0.79-0.79] |
| Bagged Tree | Acc: 0.59 [0.54-0.65]  Sen: 0.71 [0.68-0.79]  Spe: 0.32 [0.21-0.42]  AUC: 0.99 [0.97-1] | Acc: 0.58 [0.53-0.65]  Sen: 0.74 [0.68-0.82]  Spe: 0.26 [0.11-0.32]  AUC: 0.99 [0.97-1] | Acc: 0.57 [0.54-0.65]  Sen: 0.76 [0.66-0.82]  Spe: 0.24 [0.11-0.37]  AUC: 0.99 [0.95-1] | Acc: 0.76 [0.72-0.79]  Sen: 0.71 [0.63-0.76]  Spe: 0.81 [0.76-0.84]  AUC: 0.99 [0.97-1] | Acc: 0.70 [0.64-0.75]  Sen: 0.64 [0.55-0.74]  Spe: 0.76 [0.71-0.84]  AUC: 0.99 [0.97-1] | Acc: 0.71 [0.67-0.75]  Sen: 0.62 [0.55-0.68]  Spe: 0.80 [0.74-0.84]  AUC: 0.99 [0.97-1] |
| Subspace discriminant | Acc: 0.63 [0.56-0.65]  Sen: 0.86 [0.79-0.89]  Spe: 0.13 [0.11-0.26]  AUC: 0.69 [0.67-0.70] | Acc: 0.60 [0.56-0.65]  Sen: 0.84 [0.79-0.89]  Spe: 0.11 [0.1-0.15]  AUC: 0.69 [0.68-0.72] | Acc: 0.61 [0.58-0.65]  Sen: 0.82 [0.79-0.89]  Spe: 0.16 [0.11-0.26]  AUC: 0.69 [0.67-0.70] | Acc: 0.65 [0.58-0.68]  Sen: 0.66 [0.58-0.66]  Spe: 0.63 [0.58-0.71]  AUC: 0.74 [0.73-0.75] | Acc: 0.63 [0.62-0.65]  Sen: 0.61 [0.58-0.63]  Spe: 0.64 [0.63-0.68]  AUC: 0.72 [0.71-0.72] | Acc: 0.64 [0.62-0.67]  Sen: 0.66 [0.63-0.71]  Spe: 0.63 [0.61-0.66]  AUC: 0.73 [0.73-0.75] |
| Subspace KNN | Acc: 0.60 [0.56-0.65]  Sen: 0.74 [0.68-0.76]  Spe: 0.32 [0.32-0.42]  AUC: 0.69 [0.67-0.70] | Acc: 0.63 [0.60-0.70]  Sen: 0.71 [0.66-0.79]  Spe: 0.47 [0.42-0.53]  AUC: 0.69 [0.67-0.70] | Acc: 0.57 [0.51-0.63]  Sen: 0.64 [0.58-0.71]  Spe: 0.42 [0.37-0.53]  AUC: 0.69 [0.67-0.70] | Acc: 0.76 [0.68-0.80]  Sen: 0.68 [0.63-0.71]  Spe: 0.87 [0.71-0.89]  AUC: 0.69 [0.67-0.70] | Acc: 0.76 [0.72-0.79]  Sen: 0.64 [0.61-0.68]  Spe: 0.87 [0.82-0.92]  AUC: 0.69 [0.67-0.70] | Acc: 0.75 [0.72-0.76]  Sen: 0.63 [0.58-0.68]  Spe: 0.87 [0.82-0.89]  AUC: 0.69 [0.67-0.70] |
| RUSBoosted Trees | Acc: 0.65 [0.61-0.67]  Sen: 0.67 [0.58-0.71]  Spe: 0.63 [0.53-0.74]  AUC: 0.99 [0.97-1] | Acc: 0.55 [0.47-0.61]  Sen: 0.58 [0.50-0.66]  Spe: 0.47 [0.42-0.63]  AUC: 0.98 [0.97-1] | Acc: 0.58 [0.51-0.65]  Sen: 0.58 [0.47-0.66]  Spe: 0.58 [0.37-0.63]  AUC: 0.99 [0.97-1] | Acc: 0.51 [0.49-0.55]  Sen: 0.1 [0-0.1]  Spe: 0.96 [0.92-1]  AUC: 0.79 [0.79-0.96] | Acc: 0.51 [0.5-0.53]  Sen: 0.1 [0-0.1]  Spe: 0.95 [0.87-1]  AUC: 0.79 [0.79-0.97] | Acc: 0.53 [0.49-0.55]  Sen: 0.1 [0-0.1]  Spe: 0.96 [0.95-1]  AUC: 0.79 [0.79-1] |

**The LCModel control file used in this study**

$LCMODL

title= '__TITLE__'

srcraw= '__SOURCE__'

savdir= '__SAVE_DIRECTORY__'

ppmst= 4.1

ppmend= 0.2

nunfil=1024

deltat=8.334e-4

neach=99

ltable= 7

hzpppm= 123.25

filbas= ****

filtab= '__TABLE_FILE__.table'

filps= '__PS_FILE__.ps'

filraw= '__RAW_FILE__'

echot= 30.00

filcsv= '__CSV_FILE__.csv'

filcoo= '__COORD_FILE__.coord'

lcoord= 9

lcsv= 11

lprint=6

filpri= '__PRINT_FILE__.print'

WDLINE(6)=0.01

$END
